# Pain in the Cervical and Lumbar Spine as a Result of High G-Force Values in Military Pilots—A Systematic Review and Meta-Analysis

**DOI:** 10.3390/ijerph192013413

**Published:** 2022-10-17

**Authors:** Andrzej Mastalerz, Inga Maruszyńska, Krzysztof Kowalczuk, Aleksandra Garbacz, Ewelina Maculewicz

**Affiliations:** 1 Department of Biomedical Sciences, Faculty of Physical Education, Józef Piłsudski University of Physical Education in Warsaw, 00-968 Warsaw, Poland; 2 Aeromedical Training Department, Military Institute of Aviation Medicine, 01-755 Warsaw, Poland; 3 Department of Animal Genetics and Conservation, Faculty of Animal Sciences, Warsaw University of Life Sciences—SGGW, 02-787 Warsaw, Poland

**Keywords:** neck pain, lower back pain, fighter pilots, prevention, muscular loading

## Abstract

Neck pain and lower back pain are commonly reported by military pilots. That is why the answers to the following questions are important: (1) which part of the back (neck or lumbar spine) is more likely to be painful in military pilots as a result of high G-force, and (2) what intervention methods do pilots use as countermeasures for back pain resulting from high G-force? To answer these questions, the literature was searched in the following online databases: MEDLINE, PubMed, and Embase. A meta-analysis of eleven studies on pain in the neck–spine in fighter pilots vs. transport pilots showed pooled pulled OR = 1.69 (95% CI 1.25 to 2.29, I^2^ = 32%, *p*-value = 0.21); this outcome is consistent with most of the published results. A meta-analysis of five studies on pain in the lumbar spine (lower back) did not show a difference between fighter pilots vs. transport pilots with OR = 1 (95% CI 0.83 to 1.19, I^2^ = 0%, *p*-value = 0.96). The meta-analysis showed that of the two spinal segments evaluated, it was the cervical spine that showed more soreness in tactical fighter pilots. Prevention of lumbar and neck injuries should be combined with individual lumbar and neck support, as well as increasing back muscle strength through training.

## 1. Introduction

Musculoskeletal disorders are considered a primary health problem among military pilots [1]. Back pain caused by exposure to acceleration or G-force is common in military aviation [2,3,4,5,6,7,8,9]. The advent of sophisticated targeting and close-in weapon systems has not made maneuvering at high Gz force unnecessary. To maintain situational awareness in aerial combat, a fighter pilot must constantly observe the airspace. Hence, sustained high G-force causes musculoskeletal symptoms, especially in the cervical spine [6,10]. This, combined with a cramped cockpit, a largely static sitting posture, and wearing helmet-mounted equipment, can increase a pilot’s workload and cause acute or even chronic back problems. 

The army’s assessment of G-force tolerance focuses on positive G-force, including the prolonged and gradual onset of positive G-force load during centrifuge training. Positive G-force values (+Gz) are found to be associated with musculoskeletal symptoms [11] and spinal compression [12]. This information was used to develop stress suits and design flight maneuvers that would allow pilots to better tolerate high loads. Less is known about negative G-force values (-Gz). In limited studies, very low negative Gz force values have been associated with bradycardia [13]. Studies of cardiovascular changes in microgravity have demonstrated a number of adverse effects, including intravascular contracture, decreased oxygen carrying capacity [14], decreased heart rate [15], and abnormal sympathetic autonomic responses [16].

Until now, it has been hypothesized that many factors may contribute, such as unfavorable and static seating position during flight [17,18], exposure to high amplitude, low frequency vibration [19], individual physiological and biological characteristics [20], and prolonged strain on the cervical spine due to the use of the flight helmet and helmet-mounted devices [21,22,23]. 

Fighter pilots rarely consult a doctor. Research show that only 27% [6] to 43% [3] of pilots seek medical attention throughout their careers. The treatment of lumbar pain or cervical pain among fighter pilots is not widely discussed. Pilots use a variety of methods to prevent lumbar or cervical pain. These are methods used before flight, such as warming up and stretching the neck muscles as part of a “G-force warm-up” in the cockpit before exposure to high Gz force values [3,24], or in-flight, such as support of the head and neck in the cockpit during and before high Gz force values, and pre-alignment of the head before the onset of Gz force values [3,6,24]. In contrast, popular treatment methods mentioned in published studies include [3,6] rest, medication and/or physiotherapy. It has also been observed that different cockpit ergonomics can alter the predisposition to neck and lumbar pain depending on the type of aircraft [25]. 

An analysis of the literature shows that despite many studies in which military pilots are subjected to severely overloaded by high Gz forces, there is no clear indication to the answers to the following questions: (1) which part of the back (in particular, the neck or the lumbar spine) is more likely to be affected by G-force-related pain in military pilots, except helicopter pilots, and (2) which intervention methods do pilots use as countermeasures to back pain resulting from high G-force values? Therefore, the aim of the study is to answer these two research questions. This meta-analysis is the first to combine research on back pain causes among military fighter pilots as a result of high G-force values with answers to the questions of which pre-flight and in-flight behaviors or training methods can be used to counteract the effects of high G-force values’ influence.

## 2. Materials and Methods

The literature was searched using the MEDLINE, PubMed, and Embase online databases, from the inception of these databases through to 1 December 2021. The search strategy included terms suggested by the Cochrane Back Review Group [26]. The following terms were used: (jet or pilots or g force or military or fight) and (back or pain or muscles or disc or cervical or lumbar or degeneration or posture or training). The search was limited to observational studies on humans. A restriction was imposed to publications only in English and articles no older than 25 years. Case studies were also not taken for analysis.

**Figure 1:**
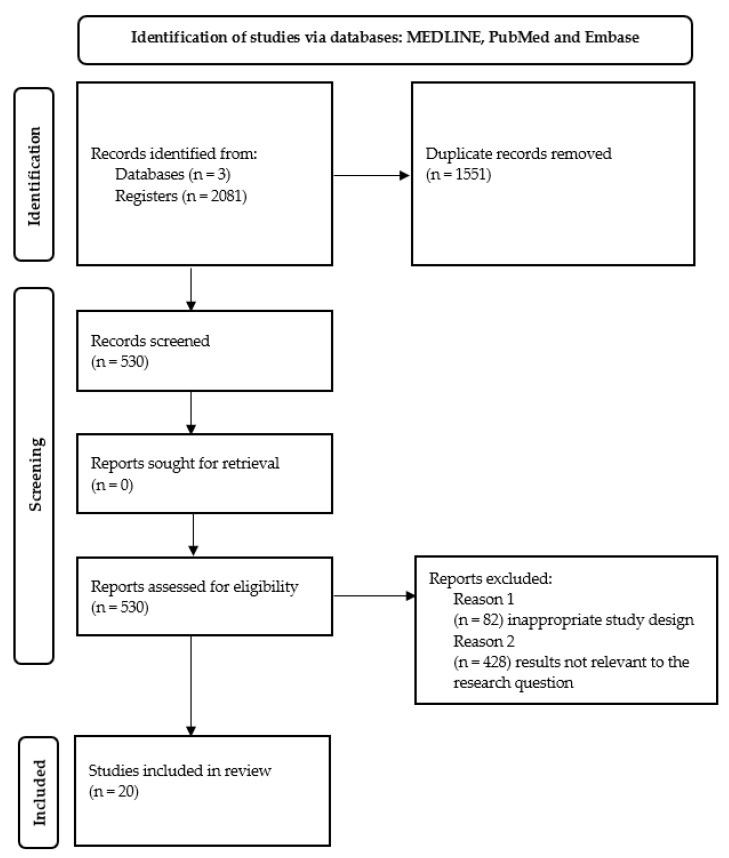
Flow chart of selection process.

The published studies were included in the systematic review if they met the following criteria: (1) a peer-reviewed original article, (2) a cohort, alternate, time series, case–control, or cross-sectional study, and (3) a study for adults aged 18 years or older. An initial keyword search yielded 2081 reports, of which 2061 were excluded due to repeat publications in the databases, for inappropriate study design, or results not relevant to the research questions (Figure 1). Additionally, due to the different ergonomics of the cabin, the articles in which helicopter pilots appeared were not included in the review. One reviewer reviewed all studies by title and excluded clearly irrelevant studies. Two reviewers independently checked all other abstracts. For each potentially eligible study, 2 reviewers independently reviewed the full text of the article and assessed whether the study met the inclusion criteria. In case of disagreement, the decision was made by consensus or, if necessary, by a third reviewer. Ultimately, 11 publications were eligible to provide the answer for the first research question and 11 publications for the second question. Two of these publications were repeated in the answers to both research questions. 

The methodological quality of the included studies was assessed using a modified Quality In Prognosis Studies tool, which was recommended by the Cochrane Prognosis Methods Group [26]. The tool assesses the risk of bias by considering 6 domains: (1) study participation, (2) study attrition, (3) prognostic factor measurement, (4) outcome measurement, (5) study confounding, and (6) approach towards statistical analysis and reporting (see Table 1 and Table 2).

Each domain contains multiple elements and is rated as having a low, moderate, or high risk of bias. The risk of bias assessment was determined for the first (Table 1) and second (Table 2) research questions separately. Two independent reviewers assessed the risk of research bias. Discrepancies were resolved by consensus, and when necessary, a third reviewer resolved disagreements.

All statistical analyses were performed using R (version 4.0.3, R Foundation for Statistics Computing, https://cran.r-project.org, accessed on 23 February 2022). The Mantel–Haenszel method was used to calculate the pulled odds ratio in one analysis. To check the heterogeneity of groups, in order to check whether a meta-analysis could be carried out, the Woolf test was used, and calculations were made using the meta function from the meta.bin package (R version 4.0.3, R Foundation for Statistics Computing, https://cran.r-project.org, accessed on 23 February 2022). A sensitivity analysis for detecting outlaying studies affecting the lack of homogeneity of groups was carried out using the Baujat graph, created using the Baujat function, and using the funnel plot created using the funnel function in the meta package (R version 4.0.3, R Foundation for Statistics Computing, https://cran.r-project.org, accessed on 23 February 2022). A forest plot was made using the forest.plot function, also from the meta package (R version 4.0.3, R Foundation for Statistics Computing, https://cran.r-project.org, accessed on 23 February 2022). 

A meta-analysis illustrated with a forest plot was chosen as the method of collective analysis of the incidence of pain in the neck spine (upper back) and pain in the lumbar spine (lower back) in fighter pilots vs. transport pilots. Meta-analysis is a statistical tool for estimating basic population effects based on a set of empirical studies on the same research question [43]. It synthesizes results from independent articles, allows us to assess the impact of the action of the analyzed factor and extends the inference from individual studies to the entire population, especially when the trials in individual experiments were small or did not achieve statistical significance. 

The chi-squared test *p*-value shown on the forest plots is the probability of the null hypothesis that there is no heterogeneity between studies, as opposed to significant heterogeneity between studies. When the *p*-value is less than 0.10, we consider that there is heterogeneity across the studies; additionally, we analyze the I^2^ statistic, which determines the percentage of observed variance that results from the actual difference in the magnitude of the studied effects. 

A random effects model was selected for analysis due to the high heterogeneity of the groups (I^2^ > 40% and *p*-value < 0.10). In this model, the cumulative effect is determined on the basis of the weighted average of the effects of individual studies; weights are determined as the inverse of the variance within the studies, and increased by the variance between the studies.

A fixed effect model was chosen when I^2^ < 40% and *p*-value > 0.10. In this model, it is assumed that the results of all studies describe the same actual effect size, and the differences in the observed effects are due to sample error. In this case, the cumulative effect is determined on the basis of the weighted average of the effects, with the weights being determined as the inverse of the variance of the respective studies.

An odds ratio (OR) was chosen as a measure of the effect under consideration due to the fact that this coefficient is used in all articles taken into account.

## 3. Results

### 3.1. G-Induced Cervical and Lumbar Pain

#### 3.1.1. Pain in the Cervical Spine

**Figure 2:**
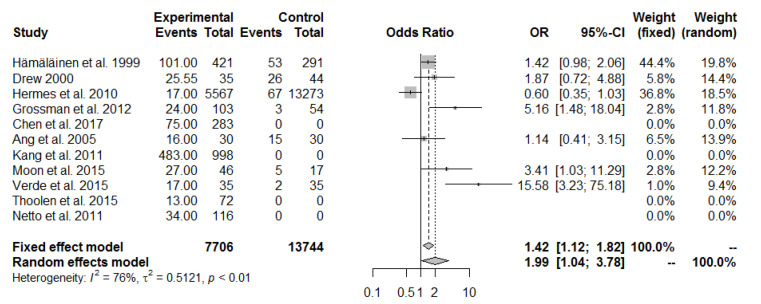
Pulled ORs of pain in the neck spine in fighter pilots vs. transport pilots. Study shows first author and year of publication; [3,27,28,29,30,31,32,33,34,35,36].

A meta-analysis of eleven studies on pain in the cervical spine in fighter pilots vs. transport pilots showed that the pooled OR was 1.99 (95% CI 1.04 to 3.78, I^2^ = 76%); this outcome is consistent with most of the published results. Only one study [28] showed a different trend (OR = 0.60, 95% CI 0.35 to 1.03). Due to I^2^ = 76% and *p*-value < 0.01, a random effect model was considered in this case (Figure 2).

**Figure 3:**
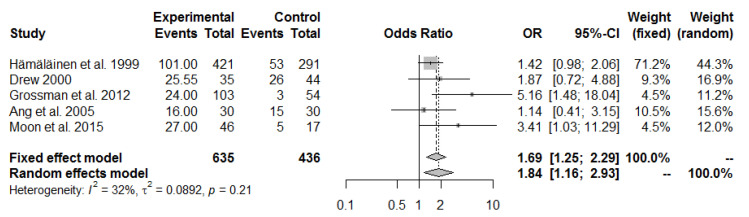
Pulled ORs of pain in the neck spine in fighter pilots vs. transport pilots. Study shows first author and year of publication; [3,27,29,31,33].

Due to the fact that I^2^ was over 75%, the funnel plot and Baujat graph were analyzed, on the basis of which several studies were removed from analysis. At first, the most outlying were chosen [34]. Then, results were cleaned from the studies where one or more groups were absent [30,32,35,36]. As a result, the pulled OR was 1.69 (95% CI 1.25 to 2.29), out of five studies. A fixed effects model was chosen due to I^2^ = 32% and *p*-value = 0.21 (Figure 3).

#### 3.1.2. Pain in the Lumbar Spine

**Figure 4:**
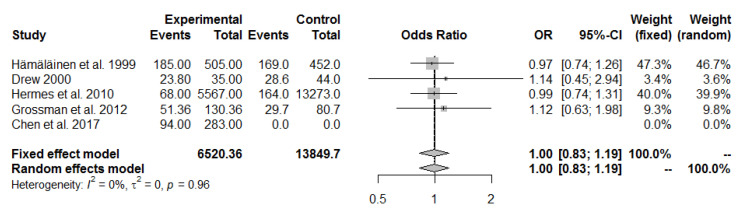
Pulled ORs of pain in the lumbar spine in fighter pilots vs. transport pilots. Study shows first author and year of publication; [3,27,28,29,30].

A meta-analysis of five studies on pain in the lumbar spine (lower back) did not show a difference between fighter pilots vs. transport pilots with OR = 1 (95% CI 0.83 to 1.19, I^2^ = 0%, *p*-value = 0.96). The two studies with the greatest weight were studies published by Hämäläinen et al. in 1999 [27] (weight = 47.3%, OR = 0.97, 95% CI 0.74 to 1.26) and published by Hermes et al. in 2010 [28] (weight = 40.0%, OR = 0.99, 95% CI 0.74 to 2.94), but neither showed a difference between pain in the lumbar spine observed in pilots depending on the aircraft type (Figure 4).

### 3.2. Prevalence of Neck and Lumbar Pain

Due to the impossibility of calculating risk indicators, as there were no reference groups for the studies conducted, this section is limited to only the description of the outcomes. The analysis presented in Table 3 indicates that some research findings recommended warming up and stretching the neck muscles as part of a “G-force warm-up” in the cockpit prior to exposure to high Gz forces [3,24]. One case showed a statistically significant beneficial effect of warm-up or isometrics [24], but others, for 95 pilots [25] and 52 pilots [6], showed no benefit. In-flight techniques include the use of external head and neck support in the cockpit during and prior to high Gz forces, and pre-positioning of the head prior to the arrival of Gz forces [3,6,24]. Two studies [3,24] showed a significant decrease in neck injuries in F-16 pilots who rested their head against the seat before the high Gz force occurred. In the case of lumbar support, neither the individualization of the level and shape of the support, [37] nor the backrest inclination angle [3] were statistically significantly effective at reducing the risk of injury to this spinal segment due to high +Gz force values. Many researchers have used different forms of strength training designed specifically for neck muscles [3,6,24], as well as whole body training including functional training or a combination of these options [3,24,25,36,37,39,40,41,42]. Whole body training including functional training or a combination of these training options has also been used for the lumbar spine [3,37,41]. Approximately half of the studies involving neck pain did not find the effects of training to be statistically significant; however, all cases of intervention methods used for the lumbar region were found to be useful (Table 3).

## 4. Discussion

Providing answers to the following two questions was the aim of this study: (1) which part of the back (neck or lumbar spine) is more likely to be in pain in military pilots as a result of high G-force, and (2) what intervention methods do pilots use as countermeasures for back pain resulting from high G-force?

### 4.1. Pain in the Cervical and Lumbar Spine

A meta-analysis of eleven studies of cervical spine pain in fighter pilots and transport pilots found that the pooled OR was 1.99 (95% CI 1.04 to 3.78, I^2^ = 76%). This confirmed that the cervical region was more painful in tactical fighter pilots, i.e., more likely to be exposed to high levels of overload. Similar results were obtained by other researchers despite their selection of helicopter pilots for comparison [44]. Even after the elimination of the studies that were missing one or more groups from this meta-analysis [30,32,35,36], an OR = 1.69 (95% CI 1.25 to 2.29) was derived from five studies. 

The results of the meta-analysis concerning the lumbar spine are entirely different. The meta-analysis of five studies on lumbar spine (lower back) pain found no difference between fighter pilots and transport pilots with OR = 1 (95% CI 0.83 to 1.19, I^2^ = 0%, *p*-value = 0.96). These results are also consistent with previous studies [44]. The cervical region of the spine has the greatest range of motion; therefore, the neck is expected to be the most stressed during high-impact maneuvers. 

Results of past studies have often shown that neck injuries are common in fighter pilots, especially those flying high-performance jet aircraft (HPJA), e.g., F-16, F-18 and F-15 [2,6,24,31,33]. Hence, it is believed that a high +Gz force value is the most important factor causing neck pain for fighter pilots. In addition, as a result of high G-force, tactical fighter pilots are more likely to experience lumbar spine pain compared to non-pilots of a similar age during their career (58% vs. 48%, respectively [27]), and reports show an overall prevalence among tactical jet pilots ranging from 25% [12,45] to 64% [27]. Musculoskeletal problems in high-performance jet aircraft pilots are ubiquitous, with an annual prevalence of neck pain in combat pilots ranging from 83% [23] to 93% [41]. The prevalence of neck and lumbar pain in the general population is approximately 37 and 15%, respectively [46,47]. Flight-related spinal disorders are classified as cervical, lumbar and thoracic pain. Kikukawa et al. [11] already found that nearly one-third of fighter pilots (with an average age of 33) had more than 10 episodes of musculoskeletal pain in their careers, and the average recovery time from one episode was eight days. According to Grossman et al. [29], fighter pilots (25%) reported more pain in multiple regions than transport pilots (9%).

In addition, aircraft type was found to be an independent predictor of clinically significant neck pain [48]. The prevalence of neck pain among fighter pilots has been studied more extensively than the prevalence of lumbar pain. Its symptoms vary depending on the type of aircraft, the age of the pilot, and the study period [31]. 

### 4.2. Prevalence of Neck and Lumbar Pain

The prevalence of spinal disorders has been widely studied in the general working-age population. Most studies have used self-reported back pain as an outcome measure, indicating that incidence rates for the general population are between 1% and 58% [49], and for the cervical spine between 0 and 58% [29]. A comparison of the prevalence of these rates in the general population suggests that fighter pilots suffer more from these symptoms. Tired muscles will be susceptible to acute injury and will not support the spine as effectively. Pilots also use strategies to counteract the effects of overload forces on the cardiovascular system, such as trunk and lower extremity muscle flexing techniques, along with pressure breathing. Together, these anti-stress strategies increase physiological tolerance [50]. However, high physiological stress is also exerted on the musculoskeletal system [38]. 

The results of the neck pain study support the postulate that headload, helmet mask, and headgear equipment combined with higher operational rate (intensity, duration, and frequency of missions) increase pilots’ pain risk [23], primarily also as a result of the pilot having to look behind the aircraft during aerial combat with the enemy in a “check-6” position that combines movement into maximum straightening, turning, and lateral bending [40,51]. Thus, the cumulative number of flight hours was identified as a significant determinant of acute musculoskeletal symptoms during flight [27,52]. It is also indicated that frequent exposure to high +Gz force causes premature disc degeneration in the cervical spine [31]. Although similar effects of lumbar degeneration have been suggested, it appears that biological processes associated with aging should also be considered as risk factors [28]. Studies in the general population have shown that the prevalence of sacral pain is highest in the third decade, and the overall prevalence increases with age until 60–65 years, and then gradually decreases [49]. 

Seating positions and seat ejection angles should also be considered when considering the musculoskeletal load on the pilot under G-force. Researchers suggest that people who sit in car seats with a back tilt of 110° to 130° for lumbar support are observed to have lower pressure on the intervertebral disc and the lowest electromyographic recordings from the spinal muscles [53]. The higher inclination (120°) of the F-16 seat negatively affected the lordosis of the cervical spine [54], which increased compressive forces on the intervertebral disc [55]. It has also been proven to affect the high prevalence of cervical conditions among F-16 pilots [25,56]. 

Evidence suggests that many pilots do not report pain or injury and continue flying for fear of losing their airworthy status [11,57,58]. Despite this, studies confirm reports of neck and lumbar pain in tactical fighter pilots during flight [39,52] or its completion [39], or during an aviation career [36,52,59,60]. Certainly, the higher incidence of neck pain in higher performance aircraft suggests that the risk of cervical spine injury is increased. Because knowledge of the severity of neck injury from high sustained +Gz force is limited, pilots are trained to take preventative measures such as: warming up neck muscles prior to G-force exposure [3,6,24,25,38], support, muscle flexing and proper neck/head positioning during exposure to high G-force [3,6,24,38], and increasing neck muscle strength [3,6,23,24,25,27,38,41]. The prevention of lumbar injuries is usually combined with individual lumbar support [3,37], as well as increased back muscle strength [3,41]. 

Fighter pilots are physically active. For example, 85% of Royal Australian Air Force fighter pilots regularly participate in some form of exercise on average three times a week [6]. Fighter pilots also consider strength training an important method to protect against flight-induced spinal disorders [11]. When comparing fighter pilots with pilots of non-high-performance aircraft, it was found that the number of hours spent on strength training per week differed between the pilots. There was no difference in hours of endurance training or other physical activity [41]. Despite this, we find that the pilots’ overall neck muscle strength was not significantly different from non-pilots, and that exposure to +Gz forces did not lead to specific changes in isometric muscle strength in any of the four major directions [61]. In the review presented in Table 3, as many as half of the publications do not observe that G-force countermeasures have a statistically significant effect. The current findings demonstrate the extent of the limitations of these research designs. Because the countermeasures to the effects of G-force used by the pilots were self-reported and not measured, only the frequency of exercise was examined. 

### 4.3. Limitation and Suggestions for Future Research

Our meta-analysis clearly indicated that out of the two spinal segments, the cervical segment shows more pain in tactical fighter pilots, i.e., it is more exposed to the effects of +Gz force. Maneuvering with a high G-force causes fatigue and results in soreness, especially in the neck, but some studies also point to the effects of this factor on the muscles of the lower back [62]. Despite various independent research efforts, no evidence has been found that it is exclusively high +Gz force that causes pain in both spinal segments.

The survey tool used in all studies concerning the prevalence of neck and lumbar pain did not require pilots to record the intensity of their training sessions. Without capturing a number of relevant training variables (frequency, intensity, timing, and type), it is difficult to assess the effectiveness of training and whether such training is associated with neck and lumbar spine pain. Future studies should attempt to capture training intensity by incorporating ratings of perceived exertion and training time into their survey instruments. Today, HIIT-compliant programs such as CrossFit, SEALFIT, and the United States Marine Corps High-Intensity Tactical Training (HITT) program are increasingly popular with military personnel [63]. The use of these programs by military pilots should become a new popular trend among military pilots to help reduce back pain.

## 5. Conclusions

Regardless of the cause, neck or lower back pain in any aviator raises concerns about health, career advancement and the associated medical and non-medical costs resulting from inability to fly. Significant pain can also be distracting or cause pilots to limit their flight maneuvers. This can greatly increase the risk of mission failure, loss/damage to the aircraft due to mishap, and/or risk to life and health. 

The meta-analysis of eleven studies of cervical spine pain in fighter pilots and transport pilots found that, out of the two spinal segments evaluated, the cervical region was more painful in tactical fighter pilots, i.e., more likely to be exposed to high levels of overload.

Fighter pilots themselves consider strength training an important method of protection against flight-induced spinal disorders. It should also be considered important for pilots to use measures to counteract high and prolonged overloads, such as warming up the neck muscles prior to G-force exposure, support, muscle tension, and proper neck/head positioning during high G-force exposure. The prevention of lumbar and neck pain should be combined with lumbar and neck support tailored to each individual, as well as increasing back muscle strength.

## Figures and Tables

**Table 1:** Risk of bias assessment for the six domains for each study for the first research question.

	Domain Number for the Risk of Bias Assessment					
First Author and Year	1	2	3	4	5	6
Hämäläinen et al., 1999 [27]	Low	Low	Low	Low	Low	Low
Drew 2000 [3]	Moderate	Moderate	Low	High	Low	Moderate
Hermes et al., 2010 [28]	Low	Low	Low	Low	Low	Low
Grossman et al., 2012 [29]	Moderate	Moderate	Moderate	Moderate	Low	Moderate
Chen et al., 2017 [30]	Moderate	Moderate	Moderate	Moderate	Low	Moderate
Ang et al., 2005 [31]	High	Moderate	Moderate	High	Low	High
Kang et al., 2011 [32]	Low	Low	Low	Low	Low	Low
Moon et al., 2015 [33]	Low	Low	Low	Low	Low	Low
Verde et al., 2015 [34]	Moderate	Moderate	Moderate	Moderate	Low	Low
Thoolen et al., 2015 [35]	High	Moderate	Low	High	Low	High
Netto et al., 2011 [36]	Moderate	Moderate	Low	Moderate	Low	Moderate

**Table 2:** Risk of bias assessment for the six domains for each study for the second research question.

	Domain Number for the Risk of Bias Assessment					
First Author and Year	1	2	3	4	5	6
Newman 1997 [6]	Low	Low	Low	Low	Low	Low
Albano et al., 1998 [24]	Moderate	Moderate	Low	High	Low	Moderate
Drew 2000, [3]	Moderate	Moderate	Low	High	Low	Moderate
Jones et al., 2000 [25]	Moderate	Moderate	Moderate	Moderate	Low	Moderate
Sovelius et al., 2008 [37]	Moderate	Moderate	Moderate	Moderate	Low	Moderate
Netto et al., 2011 [36]	Moderate	Moderate	Low	Moderate	Low	Moderate
Tucker et al., 2012 [38]	Low	Low	Low	Low	Low	Low
Wagstaff et al., 2012 [39]	40Low	Low	Low	Low	Low	Low
Lange et al., 2013 [40]	Moderate	Moderate	Moderate	Moderate	Low	Low
Rintala et al., 2015 [41]	High	Moderate	Low	High	Low	High
Rausch et al., 2021 [42]	Moderate	Moderate	Low	Moderate	Low	Moderate

**Table 3:** Characteristics of the included studies. Study shows first author and year of publication.

Author and Year	Neck/Lumbar	n/ Airplane Type	Pre-Flight Warm-Ups	In-FlightTechniques	Specific Training	Statistical Significance
Newman 1997 [6]	Neck	52/ F/A-18 Hornet, MB326H Macchi	Warming up	Neck stretches	Neck strengthening exercise	ns
Albano et al., 1998 [24]	Neck	268/ F-16	Warming up, isometrics	Head vs. seat, prepositioning the head, unloading,	Neck and body strengthening exercises	*p* < 0.05
Drew 2000 [3]	Neck/lumbar	35/ F-16 s and F-15 s	Warming up, isometrics	Neck stretches, prepositioning the head, lumbar support	Aerobic exercises, running	ns
Jones et al., 2000 [25]	Neck	95/ T-38, F-14, F-16, F/A-18	Warming up		Weight training	ns
Sovelius et al., 2008 [37]	Lumbar	11/ BAe Systems Hawk MK51		Individually shaped lumbar support		ns
Netto et al., 2011 [36]	Neck	86/ not specified			Functional strength training	*p* < 0.05
Tucker et al., 2012 [38]	Neck	82/ F/A 18	Warming up	In-flight Gz warm-up	Functional strength training	*p* < 0.05
Wagstaff et al., 2012 [39]	Neck	105/ F-16, F-5, T-38, T37, CF-104			Functional strength training	ns
Lange et al., 2013 [40]	Neck	55/ F-16			Functional strength training	*p* = 0.01
Rintala et al., 2015 [41]	Neck/ lumbar	267/ not specified			Functional strength training	ns
Rausch et al., 2021 [42]	Neck	18/ not specified			Functional strength training	*p* < 0.05

## Data Availability

Not applicable.

## References

[1] Parr J.C., Miller M.E., Pellettiere J.A., Erich R.A. (2013). Neck injury criteria formulation and injury risk curves for the ejection environment: A pilot study. Aviat. Space Environ. Med..

[2] Burnett A.F., Naumann F.L., Burton E.J. (2004). Flight-training effect on the cervical muscle isometric strength of trainee pilots. Aviat. Space Environ. Med..

[3] Drew W.E. (2000). Spinal symptoms on aviators and their relationship to G-exposure and aircraft seating angle. Aviat. Space Environ. Med..

[4] Freeman S., Karpowicz A., Gray J., McGill S. (2006). Quantifying muscle patterns and spine load during various forms of the push-up. Med. Sci. Sports Exerc..

[5] Knudson R., Macmillan D., Doucette D., Seidel M. (1988). A comparative study of G-induced neck injury in pilots of the F/A-18, A-7 and A-4. Aviat. Space Environ. Med..

[6] Newman D.G. (1997). +Gz-induced neck injuries in Royal Australian Air Force fighter pilots. Aviat. Space Environ. Med..

[7] Petrén-Mallmin M., Linder J. (2001). Cervical spine degeneration in fighter pilots and controls: A 5 year follow-up. Aviat. Space Environ. Med..

[8] Schall D.G. (1989). Non-ejection cervical spine injuries due to +Gz in high performance aircraft. Aviat. Space Environ. Med..

[9] Vanderbeek R.D. (1988). Period prevalence of acute neck injury in U.S. Air Force pilots exposed to high G forces. Aviat. Space Environ. Med..

[10] Hershkovich O., Friedlander A., Gordon B., Arzi H., Derazne E., Tzur D., Shamis A., Afek A. (2013). Associations of body mass index and body height with low back pain in 829,791 adolescents. Am. J. Epidemiol..

[11] Kikukawa A., Tachibana S., Yagura S. (1995). G-related musculoskeletal spine symptoms in Japan Air Self Defense Force F-15 pilots. Aviat. Space Environ. Med..

[12] Hämäläinen O., Vanharanta H., Hupli M., Karhu M., Kuronen P., Kinnunen H. (1996). Spinal shrinkage due to +Gz forces. Aviat. Space Environ. Med..

[13] Banks R.D., Gray G. (1994). “Bunt bradycardia”: Two cases of slowing of heart rate inflight during negative Gz. Aviat. Space Environ. Med..

[14] Buckey J.C., Gaffney F.A., Lane L.D., Levine B.D., Watenpaugh D.E., Wright S.J., Yancy C.W., Meyer D.M., Blomqvist C.G. (1996). Central Venous Pressure in Space. J. Appl. Physiol..

[15] Prisk G.K. (2014). Microgravity and the respiratory system. Eur. Respir. J..

[16] Norsk P. (2020). Adaptation of the Cardiovascular System to Weightlessness: Surprises, Paradoxes and Implications for Deep Space Missions. Acta Physiol..

[17] Thomae M.K., Porteous J.E., Brock J.R., Allen G.D., Heller R.F. (1998). Back pain in Australian military helicopter pilots: A preliminary study. Aviat. Space Environ. Med..

[18] Grant K.A. (2002). Ergonomic assessment of a helicopter crew seat: The HH-60G. Aviat. Space Environ. Med..

[19] Harrison M., Neary J., Albert W., Kuruganti U., Croll J., Chancey V., Bumgardner B. (2009). Measuring neuromuscular fatigue in cervical spinal musculature of military helicopter aircrew. Mil. Med..

[20] Visser B., van Dieen J.H. (2006). Pathophysiology of upper extremity muscle disorders. J. Electromyogr. Kinesiol..

[21] Thuresson M., Ang B., Linder J., Harms-Ringdahl K. (2005). Mechanical load and EMG activity in the neck induced by different head-worn equipment and neck postures. Int. J. Ind. Ergon..

[22] Forde K.A., Albert W.J., Harrison M.F., Neary J.P., Croll J., Callaghan J.P. (2011). Neck loads and posture exposure of helicopter pilots during simulated day and night flights. Int. J. Ind. Ergon..

[23] Lange B., Torp-Svendsen J., Toft P. (2011). Neck pain among fighter pilots after the introduction of the JHMCS helmet and NVG in their environment. Aviat. Space Environ. Med..

[24] Albano J.J., Stanford J.B. (1998). Prevention of minor neck injuries in F-16 pilots. Aviat. Space Environ. Med..

[25] Jones J.A., Hart S.F., Baskin D.S., Effenhauser R., Johnson S.L., Novas M.A., Jennings R., Davis J. (2000). Human and behavioral factors contributing to spine-based neurological cockpit injuries in pilots of high-performance aircraft: Recommendations for management and prevention. Mil. Med..

[26] Furlan A., Pennick V., Bombardier C., van Tulder M. (2009). Updated method guidelines for systematic reviews in the cochrane back review group. Spine.

[27] Hämäläinen O. (1999). Thoracolumbar pain among fighter pilots. Mil. Med..

[28] Hermes E.D., Webb T.S., Wells T.S. (2010). Aircraft type and other risk factors for spinal disorders: Data from 19,673 military cockpit aircrew. Aviat. Space Environ. Med..

[29] Grossman A., Nakdimon I., Chapnik L., Levy Y. (2012). Back symptoms in aviators flying different aircraft. Aviat. Space Environ. Med..

[30] Chen H.H., Chung C.H., Lee C.C., Yang C.S., Wen Y.S., Lee C.L., Chiang K.T. (2017). Analysis of intervertebral angulations and musculoskeletal symptoms of the spine in the military aircrews of Taiwan. Biomed. Eng. Appl. Basis Commun..

[31] Ang B., Linder J., Harms-Ringdahl K. (2005). Neck strength and myoelectric fatigue in fighter and helicopter pilots with a history of neck pain. Aviat. Space Environ. Med..

[32] Kang S., Hwang S., Lee E.T., Yang S., Park J. (2011). Measuring the cumulative effect of G force on aviator neck pain. Aviat. Space Environ. Med..

[33] Moon B.J., Choi K.H., Yun C., Ha Y. (2015). Cross-sectional study of neck pain and cervical sagittal alignment in air force pilots. Aerosp. Med. Hum. Perform..

[34] Verde P., Trivelloni P., Angelino G., Morgagni F., Tomao E. (2015). Neck pain in F-16 vs. Typhoon fighter pilots. Aerosp. Med. Hum. Perform..

[35] Thoolen S.J., van den Oord M.H. (2015). Modern air combat developments and their influence on neck and back pain in f-16 pilots. Aerosp. Med. Hum. Perform..

[36] Netto K., Hampson G., Oppermann B., Carstairs G., Aisbett B. (2011). Management of neck pain in Royal Australian Air Force fast jet aircrew. Mil. Med..

[37] Sovelius R., Oksa J., Rintala H., Siitonen S. (2008). Neck and back muscle loading in pilots flying high G(z) sorties with and without lumbar support. Aviat. Space Environ. Med..

[38] Tucker B., Netto K., Hampson G., Oppermann B., Aisbett B. (2012). Predicting neck pain in Royal Australian Air Force fighter pilots. Mil. Med..

[39] Wagstaff A., Jahr K., Rodskier S. (2012). +Gz-induced spinal symptoms in fighter pilots: Operational and individual associated factors. Aviat. Space Environ. Med..

[40] Lange B., Toft P., Myburgh C., Sjøgaard G. (2013). Effect of targeted strength, endurance, and coordination exercise on neck and shoulder pain among fighter pilots: A randomized-controlled trial. Clin. J. Pain..

[41] Rintala H., Häkkinen A., Siitonen S., Kyröläinen H. (2015). Relationships between physical fitness, demands of flight duty, and musculoskeletal symptoms among military pilots. Mil. Med..

[42] Rausch M., Weber F., Kühn S., Ledderhos C., Zinner C., Sperlich B. (2021). The effects of 12 weeks of functional strength training on muscle strength, volume and activity upon exposure to elevated Gz forces in high-performance aircraft personnel. Mil. Med. Res..

[43] Field A.P., Gillett R. (2010). How to do a meta-analysis. Br. J. Math. Stat. Psychol..

[44] Shiri R., Frilander H., Sainio M., Karvala K., Sovelius R., Vehmas T., Viikari-Juntura E. (2015). Cervical and lumbar pain and radiological degeneration among fighter pilots: A systematic review and meta-analysis. Occup. Environ. Med..

[45] Hämäläinen O., Vanharanta H., Kuusela T. (1993). Degeneration of cervical intervertebral disks in fighter pilots frequently exposed to high +Gz forces. Aviat. Space Environ. Med..

[46] Andersson G.B. (1999). Epidemiological features of chronic low-back pain. Lancet.

[47] Fejer R., Kyvik K.O., Hartvigsen J. (2006). The prevalence of neck pain in the world population: A systematic critical review of the literature. Eur. Spine J..

[48] Lawson B.K., Scott O., Egbulefu F.J., Ramos R., Jenne J.W., Anderson E.R. (2014). Demographic and occupational predictors of neck pain in pilots: Analysis and multinational comparison. Aviat. Space Environ. Med..

[49] Hoy D., Brooks P., Blyth F., Buchbinder R. (2010). The epidemiology of low back pain. Best Pract. Res. Clin. Rheumatol..

[50] Eiken O., Kölegärd R., Bergsten E., Grönkvist M. (2007). G protection: Interaction of straining maneuvers and positive pressure breathing. Aviat. Space Environ. Med..

[51] Coakwell M., Bloswick D., Moser R. (2004). High-risk head and neck movements at high G and interventions to reduce associated neck injury. Aviat. Space Environ. Med..

[52] Hämäläinen O., Vanharanta H., Bloigu R. (1994). +Gz-related neck pain: A follow-up study. Aviat. Space Environ. Med..

[53] Harrison D.D., Harrison C.O., Croft A.C., Harrison D.E., Troyanovich S.J. (1999). Sitting biomechanics part I: Review of the literature. J. Manipulative Physiol. Ther..

[54] Hoek van Dijke G.A., Snijders C.J., Roosch E.R., Burgers P.I. (1993). Analysis of biomechanical and ergonomic aspects of the cervical spine in F-16 flight situations. J. Biomech..

[55] Harms-Ringdahl K., Ekholm J., Schüldt K., Németh G., Arborelius U.P. (1986). Load moments and myoelectric activity when the cervical spine is held in full flexion and extension. Ergonomics.

[56] Hendriksen I.J., Holewijn M. (1999). Degenerative changes of the spine of fighter pilots of the Royal Netherlands Air Force (RNLAF). Aviat. Space Environ. Med..

[57] Kerstman E.L., Scheuring R.A., Barnes M.G., DeKorse T.B., Saile L.G. (2012). Space adaptation back pain: A retrospective study. Aviat. Space Environ. Med..

[58] Posch M., Schranz A., Lener M., Senn W., Äng B.O., Burtscher M., Ruedl G. (2019). Prevalence and potential risk factors of flight-related neck, shoulder and low back pain among helicopter pilots and crewmembers: A questionnaire-based study. BMC Musculoskelet. Disord..

[59] Greeves J., Wickes S., Harms-Ringdahl K. (2008). Review of the United Kingdom national work programme on the long-term effects of sustained high G on the cervical spine. NATO RTO Review of National Work Programmes on the Long-Term Effects of Sustained High G on the Cervical Spine.

[60] De Loose V., Van den Oord M., Burnotte F., Van Tiggelen D., Stevens V., Cagnie B., Danneels L., Witvrouw E. (2009). Functional assessment of the cervical spine in F-16 pilots with and without neck pain. Aviat. Space Environ. Med..

[61] Seng K.Y., Lam P.M., Lee V.S. (2003). Acceleration effects on neck muscle strength: Pilots vs. non-pilots. Aviat. Space Environ. Med..

[62] Oksa J., Hämäläinen O., Rissanen S., Salminen M., Kuronen P. (1999). Muscle fatigue caused by repeated aerial combat maneuvering exercises. Aviat. Space Environ. Med..

[63] Haddock C.K., Poston W.S., Heinrich K.M., Jahnke S.A., Jitnarin N. (2016). The benefits of high-intensity functional training fitness programs for military personnel. Mil. Med..

